# A New Imidazole Derivative for Corrosion Inhibition of Q235 Carbon Steel in an Acid Environment

**DOI:** 10.3390/polym15112420

**Published:** 2023-05-23

**Authors:** Zhongyu Huang, Lihong Liu, Bing Lei, Guozhe Meng, Zhiyuan Feng, Honglei Guo, Bokai Liao, Ping Zhang

**Affiliations:** 1 School of Chemical Engineering and Technology, Sun Yat-sen University, Zhuhai 519082, China; mc05441@connect.um.edu.mo (Z.H.); leibing@mail.sysu.edu.cn (B.L.); menggzh3@mail.sysu.edu.cn (G.M.); 2 Department of Civil and Environmental Engineering, Faculty of Science and Technology, University of Macau, Taipa, Macau, China; 3 CEPREI, Guangzhou 511370, China; gzhllh@163.com; 4 School of Chemistry and Chemical Engineering, Guangzhou University, Guangzhou 510006, China

**Keywords:** polymeric, corrosion, inhibitor, carbon steels, corrosion protection, EIS

## Abstract

Q235 carbon steel is a commonly used engineering material, but its application in marine environments is limited by its susceptibility to corrosion, especially localized corrosion that can lead to material perforation. Effective inhibitors are crucial to addressing this issue, particularly in acidic environments where localized areas become increasingly acidic. This study reports the synthesis of a new imidazole derivative corrosion inhibitor and evaluates its effectiveness in corrosion inhibition performance using potentiodynamic polarization curve and electrochemical impedance spectroscopy techniques. High-resolution optical microscopy and scanning electron microscopy were employed for surface morphology analysis. Fourier-transform infrared spectroscopy was used to explore the protection mechanisms. The results demonstrate that the self-synthesized imidazole derivative corrosion inhibitor offers an excellent corrosion protection performance for Q235 carbon steel in a 3.5 wt. % NaCl acidic solution. This inhibitor can provide a new strategy for carbon steel corrosion protection.

## 1. Introduction

Q235 carbon steel is a commonly utilized structural material in marine environments due to its favorable mechanical properties and low cost [1]. However, in marine environments, chloride ion penetration can lead to localized corrosion (such as pitting and crevice corrosion), resulting in a lower local pH due to the autocatalytic effect [2]. Therefore, it is necessary to study the anti-corrosion treatment in acidic environments.

Several methods have been explored to protect carbon steels from corrosion, including alloying [3], organic coatings [4], anodization [5,6], conversion coatings [7], and the use of inhibitors [8,9,10,11,12]. Among them, inhibitors have attracted significant attention due to their high efficiency, cost-effectiveness, and ability to provide corrosion protection without adversely affecting mechanical properties, as can be seen in the case of alloying methods [13].

Typically, corrosion inhibitors can be categorized into anodic and cathodic types. It has been reported that various corrosion inhibitors can effectively control metal corrosion in aqueous environments. Most organic compounds inhibit corrosion via adsorption on the metal substrate. The adsorption process mainly depends on the physicochemical properties of the inhibitor molecule, especially its functional groups (e.g., sulfur, oxygen, and nitrogen atom), aromaticity, possible electron density, and steric effects [14]. Most organic inhibitors adsorb to metal substrates via either physical adsorption means (such as electrostatic interactions) or chemical adsorption (such as coordination bonds). The free lone pair of electrons of the inhibitor molecules are of vital importance to corrosion inhibition [14].

Imidazole derivatives have emerged as promising corrosion inhibitors due to their high corrosion inhibition efficiency in an acidic corrosive medium and their non-toxic nature. Such organic compounds contain heteroatoms (N, O, S, and P), polar functional groups, or unsaturated bonds in their structures [15,16,17,18]. Therefore, they readily adhere to the metal surfaces with electrostatic attraction between the inhibitor and the metal (physisorption) or by transferring electrons and forming coordinate covalent bonds (chemisorption), leading to the formation of a protective layer on the metal surface that enhances the alloy’s corrosion resistance [19,20,21,22]. Imidazoline corrosion inhibitors are widely used in the petrochemical industry. It not only has a good corrosion control effect on the environment where halogen ions exist, but it also has a good corrosion inhibition effect on H_2_S and CO_2_ corrosion [14]. The reason why organic corrosion inhibitors can provide effective corrosion mitigation is that they usually contain polar functional groups, including O, N, P, and S. It is generally believed that the corrosion inhibition capabilities of the four polar functional groups follow the sequence of O < N < S < P, while their electronegativity has the opposite trend [23,24]. After studying three corrosion inhibitors containing nitrogen, oxygen, and sulfur atoms, it can be concluded that the corrosion inhibition efficiency increases in the following order O < N < S [23]. These elements are not only electronegative but also contain lone electron pairs. Polar functional groups and lone electron pairs act as adsorption centers between metals and corrosion inhibitors. Therefore, once an adsorption film is formed on the surface of the metal substrate, this film will isolate the metal from the corrosive medium, preventing the metal substrate from corrosion. The adsorption of organic corrosion inhibitors may originate from physical adsorption or chemical adsorption or the coexistence of both physical adsorption and chemical adsorption. Physical adsorption is defined as the process by which organic corrosion inhibitors and metals attract each other through electrostatic forces. On the other hand, chemisorption implies the formation of coordination bonds between the corrosion inhibitor and the metal. In addition, imidazole and imidazole derivatives have excellent corrosion inhibition performance as corrosion inhibitors in acidic media, which has been reported in previous work [23].

Despite considerable studies on corrosion inhibitors for carbon steel (such as Q235) corrosion control, fewer studies were focusing on imidazole derivatives in acidic environments. The localized corrosion of carbon steel will alter the local pH to a considerably low value. Hence, it is highly desirable to be able to identify a high-performance corrosion inhibitor in an acid environment.

In this work, we synthesized an imidazole derivative (ID) corrosion inhibitor to investigate its efficacy in preventing corrosion on Q235 carbon steel. We employed potentiodynamic polarization curves and electrochemical impedance spectroscopy (EIS) to assess the anti-corrosion performance. Additionally, scanning electron microscopy (SEM) and Fourier-transform infrared spectroscopy (FT-IR) were employed to analyze surface morphology and mechanisms, respectively. The results indicate that the self-synthesized ID corrosion inhibitor demonstrates excellent anti-corrosion effectiveness on Q235 carbon steel.

## 2. Materials and Methods

Q235 plates (0.22 wt. % C, 0.35 wt. % Si, 1.40 wt. % Mn, 0.017 wt. % P, ≤0.006 wt. % S, balance Fe) were cut into a size of 65 mm × 65 mm × 3 mm. Samples were ground using SiC paper (successively up to 2000 grit) with ethanol as the lubricant, ultrasonically cleaned in ethanol, and dried using compressed air.

**Figure 1:**
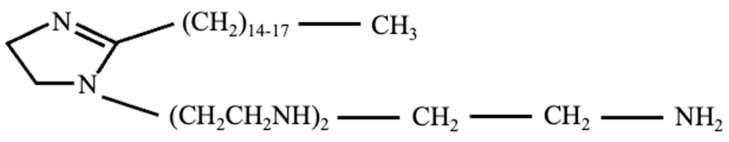
The chemical structure of self-synthesized imidazole derivative (ID).

Electrochemical experiments were carried out using a traditional three-electrode vertical cell. An area of 1 cm^2^ (face up) was exposed in the cell. A platinum plate was used as the counter electrode, and a saturated calomel electrode (SCE) served as the reference electrode. Electrochemical characterization was carried out using a Corrtest 310H (Wuhan, China) potentiostat in 3.5 wt. % NaCl solutions with additions of self-synthesized ID (the structure was shown in Figure 1) yielding a concentration ranging from 200 ppm to 1000 ppm. The pH of the solutions was adjusted to pH 3.0 using concentrated HCl. ID was synthesized under 145 °C conditions, with copper sulfate being employed as the catalyst and the solvent being cyclohexane.

All electrochemical corrosion tests were carried out using a three-electrode vertical cell with an exposure area of 1 cm^2^ (face up). The platinum plate (10 mm × 10 mm × 0.1 mm) was used as the counter electrode, and a saturated calomel electrode (SCE) served as the reference electrode. The potentiodynamic polarization measurements were measured from −0.85 V to −0.5 V vs. REF. The scan rate was 1 mV/s. All experiments were replicated a minimum of three times. EIS measurements were carried out on Q235 carbon steel in contact with testing solutions every 4 h within a 24 h time frame. All measurements were taken using a 10 mV_RMS_ sinusoidal voltage signal. Data were collected at a rate of 7 points per decade over the frequency range of 10^5^ Hz to 10^−2^ Hz.

Multiple surface characterization methods were employed in this study. High-resolution optical microscopy (Leica, Germany) was used for surface macrographs observation. SEM (Thermo Fisher Scientific, Waltham, MA, USA) was used to characterize the sample micro-morphologies. The operation voltage ranged from 3 kV to 15 kV. The functional groups of different samples were analyzed using FT-IR (PerkinElmer, Waltham, MA, USA).

## 3. Results and Discussion

### 3.1. Corrosion Inhibition Performance

The potentiodynamic polarization curve is a fast and effective corrosion evaluation method. Polarization curves can be used to better understand the electrochemical processes of metal surfaces and the changes that occur when they are subjected to changes in external environmental conditions. By extrapolating the curves, the corrosion current density of the material can be calculated. Typically, a lower corrosion current density of the material suggests a lower corresponding corrosion rate. In addition, polarization curves can also be used to study the kinetics of the anode and cathode. This helps us obtain a deeper understanding of the mechanism associated with corrosion inhibitors.

**Figure 2:**
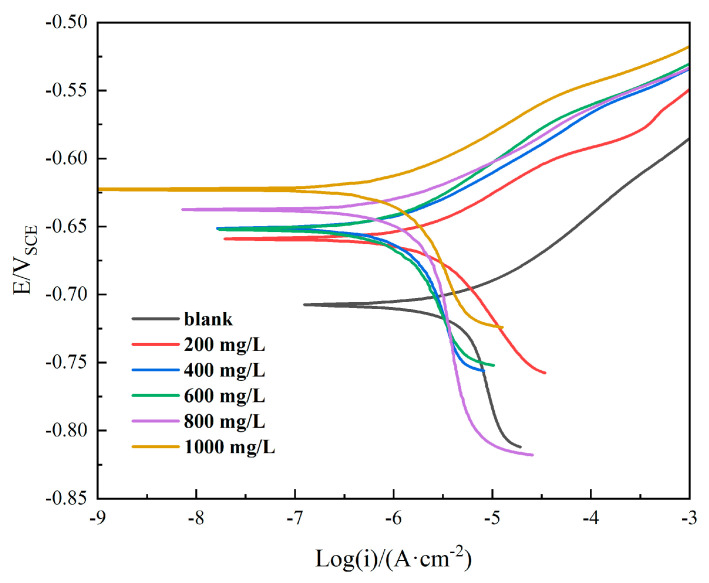
Potentiodynamic polarization curves of Q235 after 24 h immersion in different concentrations of ID.

The representative results of the potentiodynamic polarization curves of Q235 after 24 h immersion in different concentrations of ID are shown in Figure 2, including the scans resulting from curves collected without the addition of inhibitor (denoted as blank in Figure 2). All of the polarization curves obtained in ID-bearing solutions exhibited a shift to lower current densities and an increase in the open circuit potential (OCP), indicating a good corrosion inhibition on carbon steel. The increase in OCP may be attributed to the film covering on the sample surfaces [25].

After adding ID, all of the anodic polarization parts exhibited a shift to lower current densities. The active dissolution of Fe was inhibited. Except for 200 ppm and 1000 ppm, the cathodic polarization parts exhibited a shift to a lower current, indicating the cathodic oxygen reduction reaction was also inhibited. Among the six experiments, 400 ppm and 600 ppm samples behaved similarly in terms of electrochemical polarization behavior. They had similar corrosion current densities.

**Figure 3:**
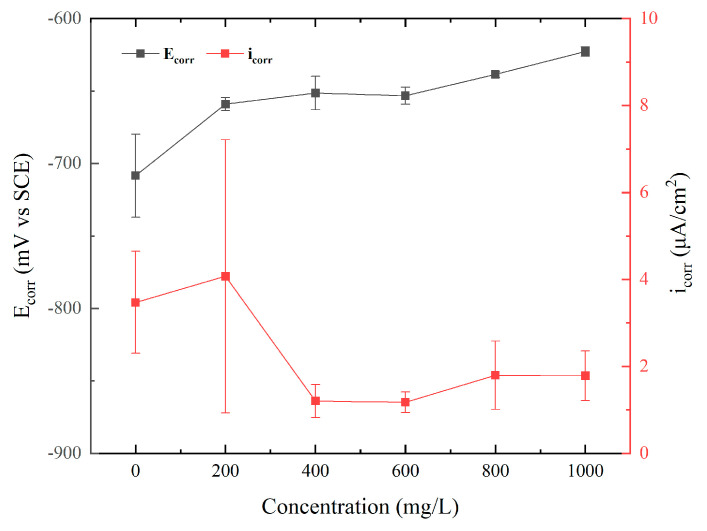
The concentration dependence of corrosion current density and corrosion potential values obtained from potentiodynamic polarization.

All experiments were replicated a minimum of three times. Figure 3 illustrates the summary of the effect of ID additions to pH 3 NaCl solution on the polarization characteristics of Q235. Values for the control experiment (without the addition of ID) were about −0.72 V_SCE_ for OCP and 3.8 × 10^−6^ A/cm^2^ for corrosion current density. Compared with the control, the addition of ID increased the OCP and decreased the corrosion current density. With the increase in the concentration of ID, the OCP increased from −0.67 V_SCE_ to −0.62 V_SCE_. According to the three replicate experiments, the corrosion current density at 200 ppm varied considerably. It was probably caused by the inhibitor being insufficiently covered on the metal surface, resulting in a less protective effect. From 200 ppm to 1000 ppm, the corrosion current density values were similar and ranged from 1 × 10^−6^ A/cm^2^ to 4.2 × 10^−6^ A/cm^2^. Overall, 400 ppm and 600 ppm had very similar inhibition performances, and these results were better than others. According to Figure 3, the 600 ppm scenario had a more stable electrochemical behavior, and it was slightly better than that of the 400 ppm scenario. Therefore, 600 ppm was chosen for the following tests.

EIS, also known as AC Impedance Spectroscopy, is a method used to analyze the kinetic characteristics and electricity of the electrochemical interface by applying an alternating voltage to the electrochemical system and measuring the amplitude ratio and phase difference between the voltage and current. The basis of the EIS principle is the electrical and kinetic properties of electrochemical reactions. In an electrochemical reaction, electrons and ions are transferred across the electrode surface to form an electrochemical interface. The properties of this interface determine the efficiency and rate of the electrochemical reaction. In an electrochemical system, there is a certain phase difference and amplitude ratio between current and voltage. This is due to the kinetic and electrical characteristics of the electrochemical reaction. By measuring these parameters (the impedance of the electrochemical interface), the properties of the electrochemical interface can be understood. As an important electrochemical analytical method, EIS provides a reference for the design and optimization of electrochemical systems. It can be used to study various electrochemical systems, including batteries, electrolytes, and electrodes. In addition to being used to study the kinetics and electrochemical characteristics of electrochemical reactions, it can also provide deeper insights with respect to the mechanism and influencing factors of electrochemical reactions.

**Figure 4:**
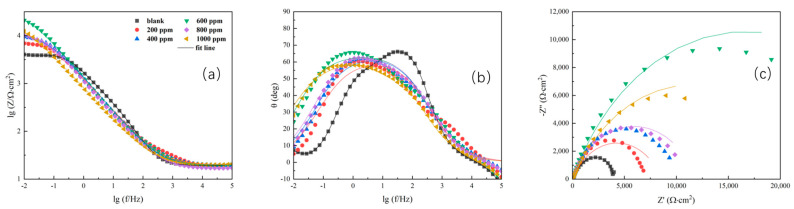
Bode plots (**a**,**b**) and Nyquist plot (**c**) results of various concentrations of ID in acid 3.5 wt. % NaCl solution after immersion for 24 h.

**Figure 5:**
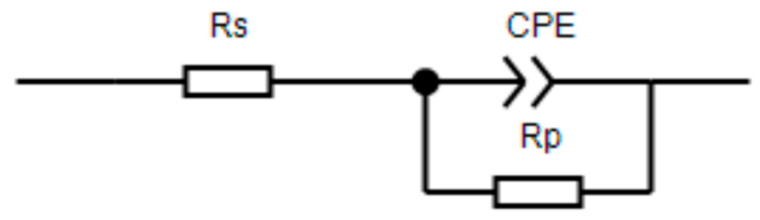
The equivalent circuit model [34].

Generally speaking, in the study of corrosion inhibitors, the low-frequency impedance value can be used for a rapid evaluation of the effect of corrosion inhibitors. A higher low-frequency impedance value is indicative of greater resistance to charge transfer, which means a slower corrosion rate of the material. Further EIS analysis can be conducted using EIS curve-fitting. The interface is simulated using different equivalent circuits. Through the analysis of the EIS plots, the protection mechanism of the corrosion inhibitor can be proposed. EIS can be carried out to study electrochemical processes at the interface and has been successfully adopted to evaluate the corrosion resistance of different coatings [26]. Figure 4 shows EIS Bode and Nyquist spectra after immersion in the different concentrations of ID solutions for 24 h. The spectra all fit the equivalent circuit model shown in Figure 5, which is a classic model for organic inhibitors [27]. R_s_ represents the solution resistance. R_p_ is the polarization resistance. CPE is the interfacial reactance that develops due to charge separation at the interface [27,28].

According to Figure 4a–c, the Bode and Nyquist plots indicate a classic interface model that matches the equivalent circuit model in Figure 5. The main protection mechanism of the ID inhibitor was absorption [29]. The corrosion inhibitor is adsorbed on the sample surface resulting in a charge separation at the interface. Adsorbed corrosion inhibitors can provide resistance to the penetration of aggressive ions [25,30,31,32,33]. According to the value of polarization resistance (R_p_), the solution with ID clearly increased the impedance. Among different ID concentrations, 600 ppm showed the highest impedance value at the low-frequency range.

**Figure 6:**
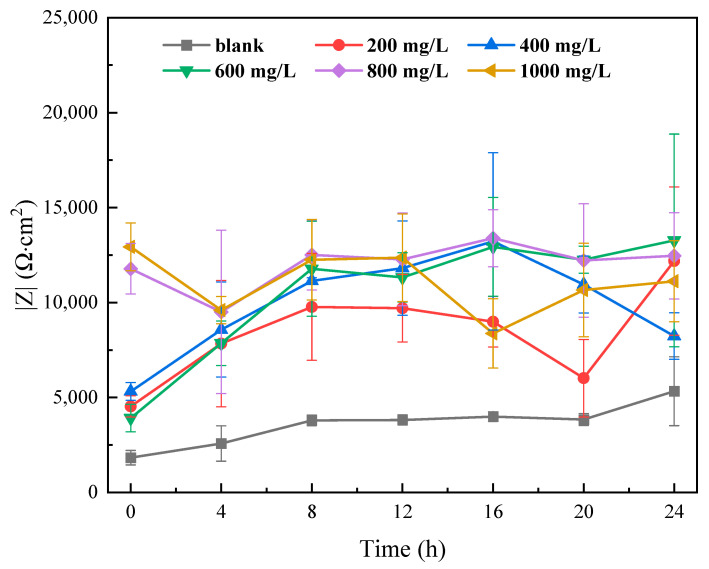
Polarization resistance (R_p_) summary of various concentrations of ID in an acidic 3.5 wt. % NaCl solution. Measurements were obtained in 4 h intervals.

The inhibitor protection effect can be estimated with the value of R_p_. To facilitate comparison, the dependence of R_p_ measured by EIS on ID concentrations was shown in Figure 6. The addition of ID increased the anti-corrosion performance of Q235, as evidenced by the increase in R_p_. From 200 ppm to 1000 ppm, the overall performance of 200 ppm was the worst. The relatively low inhibitor concentration (200 ppm) was not able to provide good protection and was caused by an insufficient covering on the sample surface. However, an exceedingly high concentration of corrosion inhibitor (1000 ppm) was not providing desirable protection, which was caused by the steric hindrance effect. Scenarios of 600 ppm and 800 ppm had a similar trend within 24 h of EIS monitoring, with 600 ppm showing a higher R_p_ value after 24 h immersion. The EIS results had a good match with the potentiodynamic polarization test, which all indicated 600 ppm was the best concentration.

### 3.2. Characterization

**Figure 7:**
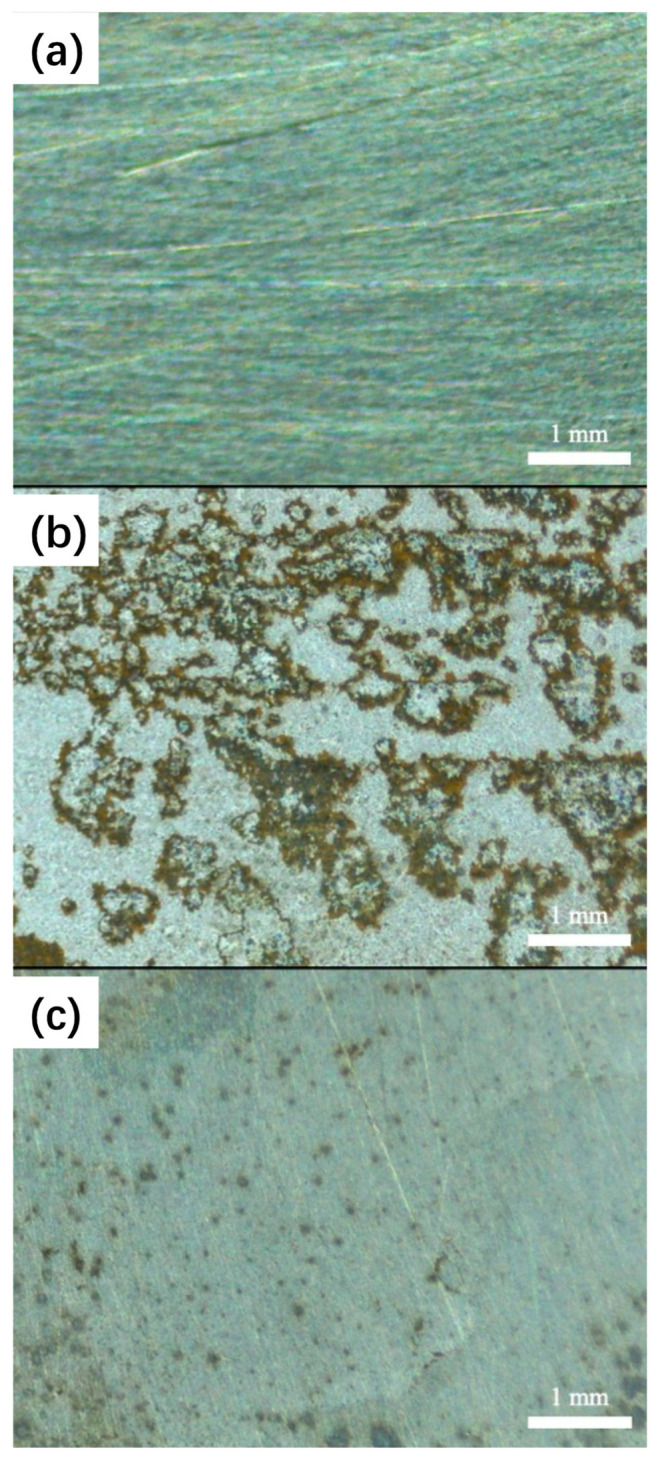
The optical micrographs obtained from the surface of Q235: without immersion test (**a**); after immersion in pH 3 NaCl solutions for 24 h (**b**); after immersion in pH 3 NaCl solution with 600 ppm ID (**c**). All samples were ultrasonically cleaned before testing.

According to the electrochemical corrosion test results, the 600 ppm ID inhibitor behaved with the best anti-corrosion performance. Therefore, the concentration of ID was selected as 600 ppm in the following studies. In Figure 7, the optical micrographs were obtained from the surface of Q235, with the polished sample acting as a reference (Figure 7a). Without corrosion inhibitors, the surface of Q235 was corroded significantly (Figure 7b). The grey-colored corrosion products were observed on the surface. In comparison, the sample with 600 ppm ID inhibitor was clean, with only some small grey dots observed on the surface (Figure 7c). From the prospect of surface morphologies, ID inhibitor has an apparent protective effect.

**Figure 8:**
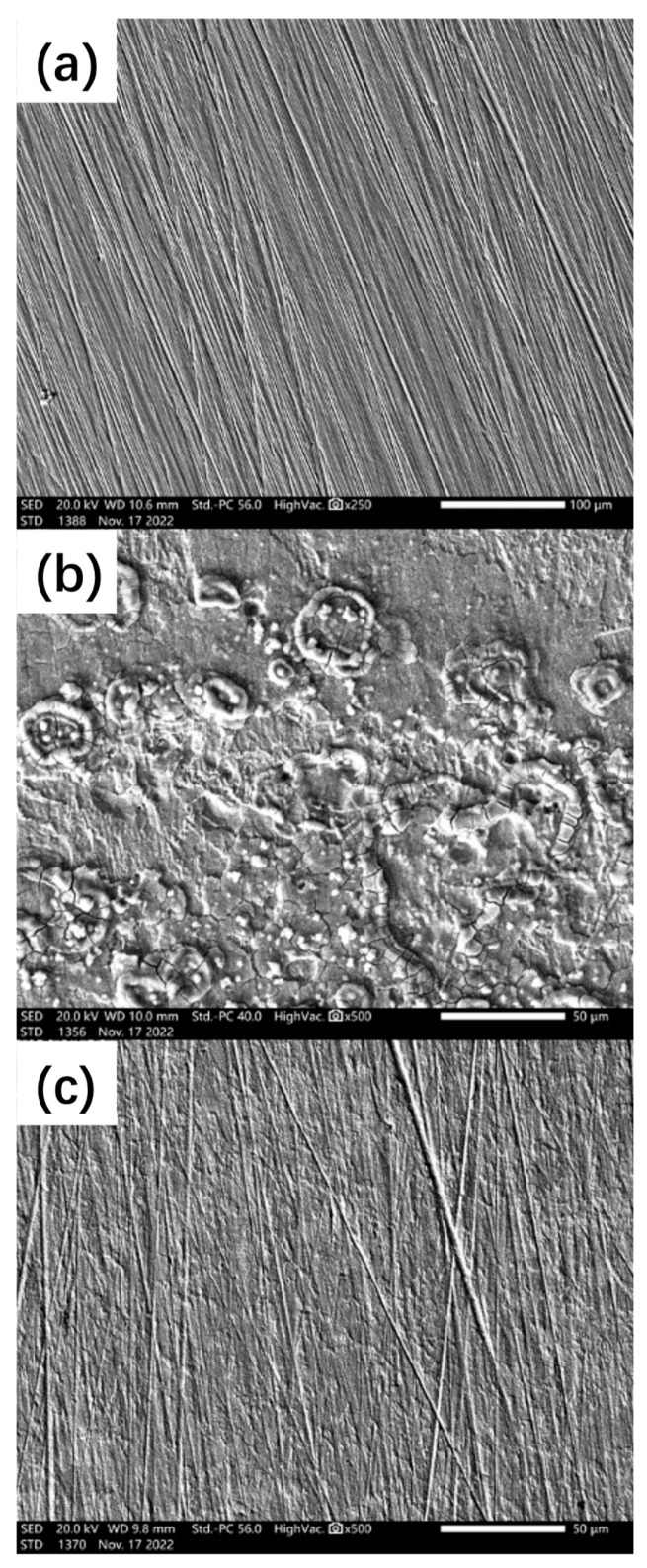
The SEM micrographs obtained from the surface of Q235: without immersion test (**a**); after immersion in pH 3 NaCl solutions for 24 h (**b**); after immersion in pH 3 NaCl solution with 600 ppm ID (**c**).

More detailed surface morphologies were obtained using SEM (Figure 8), with the polished sample serving as a reference (Figure 8a). The scratches on the surface were due to the surface polishing. In Figure 8b, substantial corrosion products were observed on the metal surface. Additionally, there were no polishing scratches observed on the surface, indicating that the top metal top layer was corroded or that the corrosion products were covered on the surface. The addition of 600 ppm ID improved the corrosion resistance of the steel, which was evidenced by Figure 8c. No obvious corrosion products were found, and the polishing scratches were clearly observed. The surface morphology analysis in Figure 7 and Figure 8 provided strong evidence that the self-synthesized ID has a significant corrosion protection effect.

**Figure 9:**
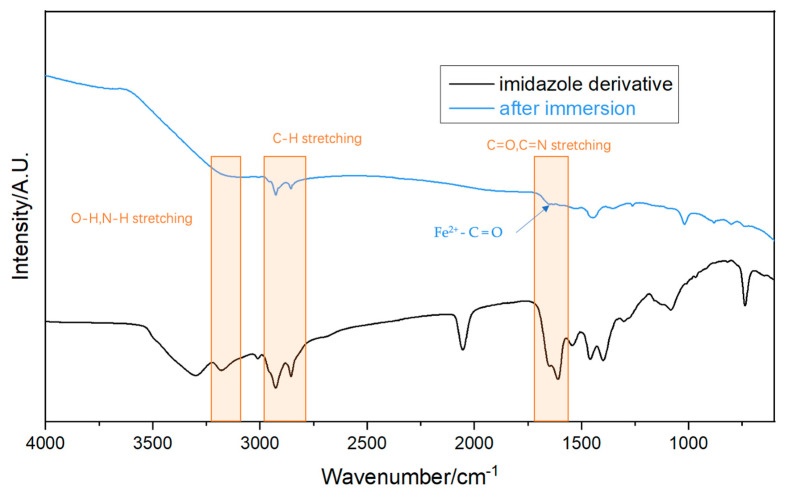
FT-IR spectra of ID and Q235 after 24 h immersion in pH 3 NaCl solution with 600 ppm ID.

To study the protection mechanism of self-synthesized ID on Q235, the FT-IR test was employed (Figure 9). The bands between 2800 cm^−1^ and 2938 cm^−1^ correspond to symmetric stretching vibrations of C-H. From 3100 to 32,500 cm^−1^, the bands may be attributed to the O-H and N-H stretching vibration. The stretching vibration of C=O and C=N is located around 1730 cm^−1^ [35]. After immersion in pH 3 NaCl solution with 600 ppm ID, the surface of Q235 was measured with FT-IR. Many bands had a good match with ID. The protective film consists of Fe^2+^–C=O group and N–H stretch on the metal surface, indicating the adsorption of ID [36]. The FTIR spectra study reveals that the protective film on Q235 was caused by the adsorption of ID. Since the metal surface was occupied with ID, the intrusion of Cl^−^, O_2_, and H_2_O was inhibited [11]. Hence, the Q235 substrate was well-protected with excellent corrosion resistance. The ID inhibitor formed a protective layer on the surface of Q235 carbon steel, effectively preventing the intrusion of corrosive species such as Cl^−^, O_2_, and H_2_O to the metal surface. The main protection mechanism was attributed to its adsorption onto the metal surface, leading to the formation of a stable protective layer.

It can be concluded that the adsorption of ID was able to provide excellent corrosion protection on the carbon steel surface under an acidic environment. This is particularly significant for the inhibition of the growth of localized corrosion on carbon steel since the pH is low in corrosion pits. This inhibitor can provide a new strategy for carbon steel corrosion protection. In comparison with azathione derivatives, the self-synthesized ID inhibitor behaved with a better overall protection effect [37]. For future studies, the self-synthesized ID must be tested in different temperatures to explore the possible applications in different environments.

## 4. Conclusions

The present study focused on evaluating the effectiveness of a self-synthesized imidazole derivative (ID) corrosion inhibitor for the protective effect on Q235 carbon steel against localized corrosion in an acid 3.5 wt. % NaCl solution. The addition of 600 ppm ID resulted in a significant decrease in corrosion current density from 3.8 × 10^−6^ A/cm^2^ to 1 × 10^−6^ A/cm^2^, indicating excellent corrosion inhibition performance. Moreover, the value of R_p_ increased from 4700 Ohm × cm^2^ to 13,000 Ohm × cm^2^, further confirming the superior anti-corrosion properties of ID inhibitor. Additionally, the surface morphology analysis revealed that the ID inhibitor formed a protective layer on the surface of Q235 carbon steel, effectively preventing the intrusion of corrosive species such as Cl^−^, O_2_, and H_2_O to the metal surface. The main protection mechanism was attributed to its adsorption onto the metal surface, leading to the formation of a stable protective layer.

## Data Availability

The data presented in this study are available on request from the corresponding author.
